# Benefit and risk of adding rivaroxaban in patients with coronary artery disease: A systematic review and meta‐analysis

**DOI:** 10.1002/clc.23514

**Published:** 2020-11-21

**Authors:** Cheng Xie, Yongfu Hang, Jianguo Zhu, Caiyun Li, Bin Jiang, Yuzhen Zhang, Liyan Miao

**Affiliations:** ^1^ Department of Clinical Pharmacology the First Affiliated Hospital of Soochow University Suzhou China; ^2^ Department of Pharmacy the Affiliated Suzhou Science and Technology Town Hospital of Nanjing Medical University Suzhou China; ^3^ Department of Cardiology the First Affiliated Hospital of Soochow University Suzhou China

**Keywords:** coronary artery disease, meta‐analysis, rivaroxaban

## Abstract

**Background:**

Although the European Medicines Agency and the US Food and Drug Administration have, respectively, approved rivaroxaban for the prevention of recurrent major adverse cardiovascular events in patients with myocardial infarction and stable coronary artery disease, its efficacy and safety is unclear. This meta‐analysis aimed to evaluate the benefit and risk of adding rivaroxaban in coronary artery disease (CAD) patients, focusing on treatment effects stratified by different baseline clinical presentations.

**Hypothesis:**

There are differences in treatment effects of adding rivaroxaban among CAD patients with different baseline clinical presentations.

**Methods:**

Medline, EMBASE, and Cochrane Databases were systematically searched from inception to 21 July 2020 for randomized controlled trials (RCTs) comparing rivaroxaban in CAD patients. The primary efficacy endpoint and safety endpoint were assessed by using Mantel–Haenszel pooled risk ratios (RRs) and 95% confidence intervals (CIs).

**Results:**

Five RCTs that included 43 650 patients were identified. Patients receiving rivaroxaban had a significantly lower risk of the primary efficacy endpoint (RR, 0.86; 95% CI, 0.76–0.97, *p* = .01) accompanied by increased risk of the primary safety endpoint (RR, 1.83; 95% CI, 1.10–3.05, *p* = .02). Subgroup analyses showed that in males the risk–benefit appears to be more favorable while in patients ≥65 years, in females, in patients with diabetes, those with mild to moderate impaired renal function, and region of Asia/other seems unfavorable.

**Conclusion:**

Rivaroxaban may provide an additional choice for secondary prevention in CAD patients. However, careful estimation of the risk of ischemic and bleeding events using patient characteristics are critical to achieving net benefit.

## 1 INTRODUCTION

Antiplatelet agents are the cornerstone of secondary prevention in patients with coronary artery disease (CAD). Guidelines recommend lifelong use of single antiplatelet therapy in all patients with stable coronary artery disease (SCAD) and dual antiplatelet therapy (DAPT) in patients following acute coronary syndrome (ACS) for 12 months.^1^ Despite the adherence to recommended antiplatelet therapy (APT), 12.2% of patients with SCAD and 18.3% of patients with ACS experience recurrent major adverse cardiovascular events (MACE).^2^ There is evidence that anticoagulation is effective in reducing ischaemic events in ACS during the acute phase and that the combination with platelet inhibitors is more effective than either treatment alone.^3^, ^4^

Early meta‐analyses revealed adding direct oral anticoagulants (DOAC) to APT in ACS after the acute phase could reduce the risk of ischemic events at the cost of a higher risk of bleeding.^5^, ^6^ However, with the results of the ATLAS ACS 2‐TIMI 51 trial,^7^ the European Medicines Agency (EMA) approved rivaroxaban 2.5 mg twice daily for non‐ST‐elevation myocardial infarction and ST‐segment elevation myocardial infarction (STEMI) patients after the acute phase.^8^ Subsequently, the US Food and Drug Administration (FDA) approved rivaroxaban for the prevention of recurrent adverse cardiovascular events in patients with SCAD according to the results of the COMPASS trial.^9^

Nonetheless, recently meta‐analyses demonstrated the addition of rivaroxaban to APT regimen was effective in patients with CAD, but the safety outcome was doubtful.^10^, ^11^, ^12^ Interestingly, Chiarito et al^13^ found the risk–benefit profile of DOAC appears unfavorable in patients with NSTE‐ACS, whereas DOAC in addition to APT might represent an attractive option for patients with STEMI. Therefore, we conducted a meta‐analysis to evaluate the benefit and risk of adding rivaroxaban in patients with CAD and focusing on treatment efficacy and safety stratified by different baseline clinical presentations.

## 2 METHODS

We systematically searched Medline, EMBASE, and Cochrane Databases for all relevant articles adding rivaroxaban in patients with coronary heart disease through 21 July 2020. The literature was searched with the following keywords: Rivaroxaban, anticoagulant, coronary artery disease, coronary heart disease, acute coronary syndrome and random*. A comprehensive search of reference lists of all review articles and original studies retrieved by this method was performed to identify additional studies.

### 2.1 Inclusion and exclusion criteria

Inclusion criteria were the following: (1) trials designed as RCT; (2) trials based on patients with CAD, including SCAD and ACS; (3) trials compared outcomes which were observed with the addition of rivaroxaban to APT; (4) trials reported the primary efficacy endpoint (ischemic events) and/or safety endpoint (bleeding events). Exclusion criteria were the following: (1) trials included patients need continued or planned treatment with rivaroxaban, such as atrial fibrillation and pulmonary embolism; (2) trials with sample size less than 500 or follow‐up <6 months; (3) duplicate reports.

### 2.2 Data abstraction

Two investigators (Cheng Xie and Yongfu Hang) independently assessed studies for possible inclusion by reading titles and/or abstracts, then viewed the full‐texts of the remaining publications to pick up the ultimately available studies. Data extraction was done by one reviewer (Cheng Xie), and subsequently cross‐checked by the other reviewer (Yongfu Hang). Any divergences were discussed or determined by a third investigator (Jianguo Zhu). Following information was abstracted: the first author and publication year, inclusion and exclusion criteria, data source, sample size, baseline features of patients, treatment features, follow‐up time, the primary endpoints and their definitions.

### 2.3 Bias risk and study quality assessment

The methodological quality of eligible studies was assessed by the Cochrane collaboration's tool for assessing risk of bias including the following criteria: sequence generation, allocation concealment, blinding, incomplete outcome data, selective outcome reporting, and other issues. The bias risk of each study was scored as low, unclear, or high in each section.

### 2.4 Statistical analysis

Dichotomous data were expressed as RR with 95% confidence intervals (CIs). Heterogeneity of effect size across the studies was tested using Q statistics at the *p* < .10 level of significance. We also calculated the I^2^ statistic with a quantitative measure of inconsistency across the studies. The data were pooled by random‐effects model in case significant heterogeneity (Cochran test with *p* < .10 or I^2^ > 50%) was found. Otherwise, the fixed‐effects model was used. Sensitivity analyses with fixed‐effect models were performed to assess consistency among effect estimates that were obtained with random‐ and fixed‐effects models. Potential publication bias was visually inspected by funnel plot if more than 10 studies. We conducted subgroup analyses according to age (< 65 and ≥ 65 years), sex (male and female), history of previous myocardial infarction (yes and no), history of diabetes (yes and no) renal function (mild to moderate impaired and moderate to severe impaired) and geographic region (America, Europe and Asia/other). Meta‐analysis was performed with the software of Cochrane Review Manager 5.1.2 (Cochrane Library Software, Oxford, UK).

## 3 RESULTS

**FIGURE 1:**
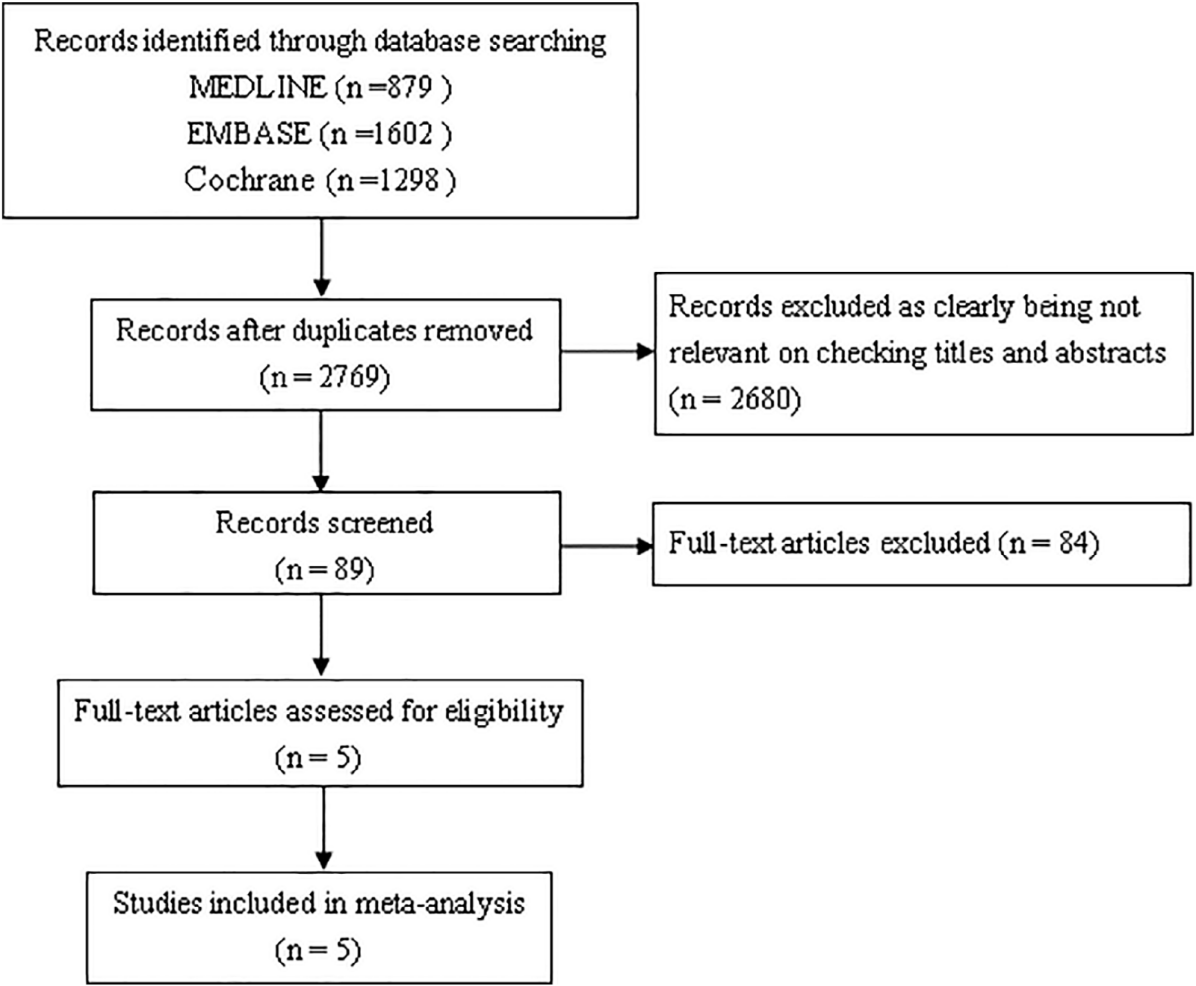
Study selection according to the PRISMA model

Figure 1 shows a flow diagram for the selection process. A total of five RCTs^7^, ^9^, ^14^, ^15^, ^16^ that included 43 650 patients were finally identified. Table 1 summarizes the characteristics of the selected studies. There were three studies^7^, ^14^, ^16^ compared rivaroxaban with placebo and two studies^9^, ^15^ compared rivaroxaban with aspirin. Among the five RCTs, three studies^7^, ^14^, ^15^ were ACS patients and the other two studies were SCAD patients^9^ and CAD patients,^16^ respectively. The methodological quality of the included studies was, in general, good as shown in Table 1.

**TABLE 1:** Characteristics of the included studies

Study name	Publication year	NCT number	Period of study	Country	Center	Study patients	Number of patients (I/C)	Mean age (I/C, years)	Male (I/C, %)	Follow‐up (months)
ATLAS ACS‐TIMI 46^14^	2009	00402597	2006–2008	27	297	ACS	2331/1160	57.2/57.8	77.6/76.3	6
ATLAS ACS 2–TIMI 51^7^	2012	00809965	2008–2011	44	766	ACS	10 350/5176	61.9/61.5	74.6/75.0	13.1
GEMINI‐ACS‐1^15^	2017	02293395	2015–2016	21	321	ACS	1519/1518	62/63	75/75	11
COMPASS^9^	2017	01776424	2013–2016	33	602	SCAD	8313/8261	69/69	79/80	23
COMMANDER HF^16^	2018	01877915	2013–2017	32	628	CAD	2507/2515	66.5/66.3	78.0/76.2	21

Study name	Intervention	Control	Primary efficacy endpoint	Primary safety endpoint	Study quality
ATLAS ACS‐TIMI 46^14^	Rivaroxaban (5–20 mg)	Placebo	Death, MI, stroke, or severe recurrent ischaemia requiring revascularisation	Major bleeding, minor bleeding, or bleeding requiring medical attention (TIMI)	Low risk
ATLAS ACS 2–TIMI 51^7^	Rivaroxaban (5–10 mg)	Placebo	CV death, stroke or MI	Major bleeding not related to CABG (TIMI)	Low risk
GEMINI‐ACS‐1^15^	Rivaroxaban (5 mg)	Aspirin	CV death, MI, stroke, or definite ST	Non‐CABG surgery clinically significant bleeding (TIMI)	Low risk
COMPASS^9^	Rivaroxaban (5 mg)	Aspirin	CV death, stroke or MI	Major bleeding and included fatal bleeding, symptomatic bleeding into a critical organ, bleeding into a surgical site requiring reoperation, and bleeding that led to hospitalization (ISTH)	Low risk
COMMANDER HF^16^	Rivaroxaban (5 mg)	Placebo	Death, MI, or stroke	Fatal bleeding or bleeding into a critical space with a potential for causing permanent disability (ISTH)	Low risk

Abbreviations: ACS, acute coronary syndromes; C, control group; CABG, coronary artery bypass grafting; CAD, coronary artery disease; CV, cardiovascular; I, intervention group; ISTH, international society on thrombosis and hemostasis; MI, myocardial infarction; SCAD, stable coronary artery disease; ST, stent thrombosis; TIMI, thrombolysis in myocardial infarction.

**FIGURE 2:**
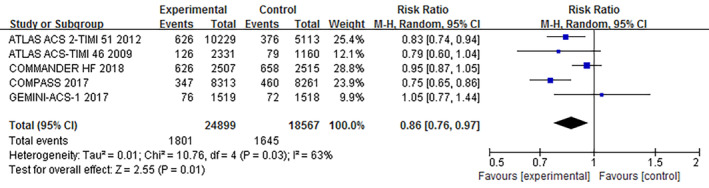
Meta‐analysis for the primary efficacy endpoint

**FIGURE 3:**
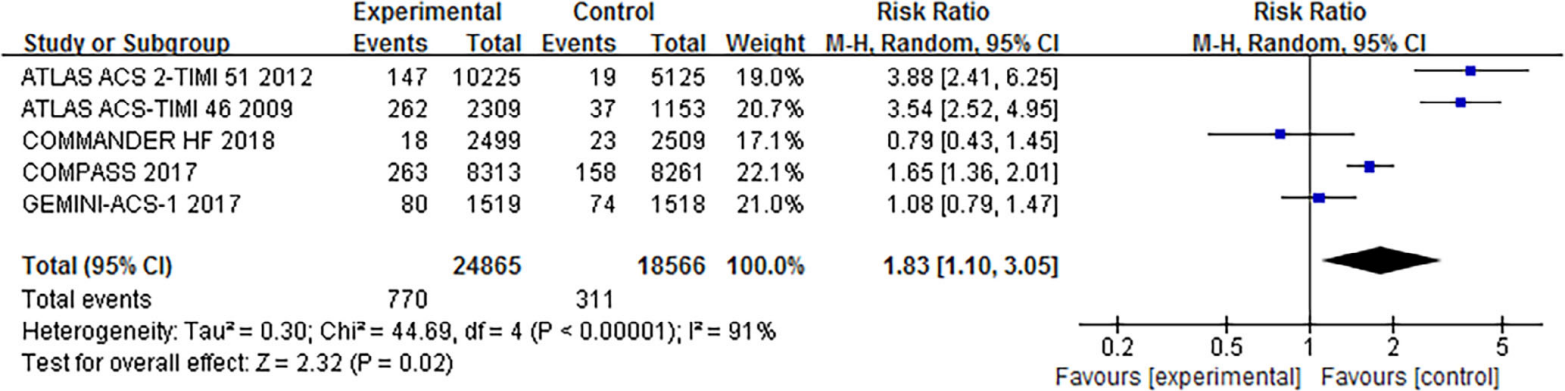
Meta‐analysis for the primary safety endpoint

The primary efficacy endpoint and safety endpoint were those adopted by the original studies. Our pooled analysis indicated that addition of rivaroxaban significantly reduced the incidence of the primary efficacy endpoint (RR, 0.86; 95% CI, 0.76–0.97, *p* = .01) (Figure 2). However, addition of rivaroxaban was associated with significantly higher risk of the primary safety endpoint (RR, 1.83; 95% CI, 1.10–3.05, *p* = .02) (Figure 3).

To explore the study heterogeneity, we further performed meta‐analysis in subgroups based on several baseline clinical presentations (age, sex, history of MI, diabetes, renal function and region). Table 2 shows the risk of the primary efficacy endpoint in patients ≥65 years (RR, 0.81; 95% CI, 0.68–0.96, *p* = .02), in males (RR, 0.85; 95% CI, 0.75–0.96, *p* = .008), in patients without diabetes (RR, 0.82; 95% CI, 0.74–0.91, *p* = .0002), those with mild to moderate impaired renal function (RR, 0.89; 95% CI, 0.82–0.98, *p* = .01), America patients (RR, 0.78; 95% CI, 0.67–0.90, *p* = .0006) and Europe patients (RR, 0.85; 95% CI, 0.74–0.97, *p* = .02),which was significantly reduced in case of rivaroxaban treatment compared to control group. A similar risk of the primary efficacy endpoint was observed in patients with and without history of MI (RR, 0.86; 95% CI, 0.80–0.93, *p* = .0002 and RR, 0.85; 95% CI, 0.76–0.94, *p* = .002, respectively). The risk of primary efficacy endpoint in patients < 65 years, in females, in patients with diabetes, those with moderate to severe impaired renal function, and Asian/other patients, which was reduced in case of rivaroxaban treatment compared to control group, but not significantly. As for the risk of the primary safety endpoint, only in males subgroup did not significantly increase (RR, 2.28; 95% CI, 0.97–5.35, *p* = .06).

**TABLE 2:** Subgroup analyses for the primary efficacy endpoint and safety endpoint (RR, 50%Cl)

Subgroup		The primary efficacy endpoint	The primary safety endpoint
Age	<65y	0.84 (0.70–1.02)	2.28 (1.17–4.47)
	≥65y	0.81 (0.68–0.96)	3.26 (1.42–7.51)
Sex	Male	0.85 (0.75–0.96)	2.28 (0.97–5.35)
	Female	0.86 (0.66–1.10)	3.01 (1.34–6.74)
History of MI	Yes	0.86 (0.80–0.93)	2.79 (1.23–6.31)
	No	0.85 (0.76–0.94)	2.79 (1.63–4.78)
Diabetes	Yes	0.89 (0.76–1.05)	2.83 (1.35–5.89)
	No	0.82 (0.74–0.91)	2.67 (1.50–4.74)
Renal function	Mild to moderate impaired	0.89 (0.82–0.98)	3.41 (2.56–4.54)
	Moderate to severe impaired	0.94 (0.83–1.06)	7.47 (2.34–23.82)
Region	America	0.78 (0.67–0.90)	3.08 (1.18–8.04)
	Europe	0.85 (0.74–0.97)	3.00 (1.35–6.69)
	Asia and other	0.86 (0.66–1.22)	2.23 (1.60–3.11)

There was no difference in the results between the fixed‐effect model and the random‐effect model for the primary efficacy and safety endpoint.

## 4 DISCUSSION

Plaque rupture and thrombosis are the major concerns in patients with CAD. Platelet adherence to subendothelial components triggers a number of amplification pathways required for the formation of a stable thrombus. Soluble agonists, like thromboxane and adenosine diphosphate, are the main amplifiers of platelet activation and are the targets of the most prescribed antiplatelet drugs.^17^ On the other hand, recurrent cardiovascular events may be related to persistent elevation of thrombin beyond the index event,^18^ which leads to progression of cardiovascular disease by inducing inflammation, endothelial dysfunction and thrombosis.^19^ Thrombin is also a potent platelet activator, and could therefore promote thrombus formation in this way. In fact, activation of primary hemostasis and coagulation are two closely related events that together contribute to thrombus formation, since activated platelets support the coagulation cascade by providing a negatively charged scaffold, as well as thrombin being a potent platelet activator. Therefore, patients with CAD should receive antiplatelet combined anticoagulation therapy theoretically.

In this meta‐analysis, we assessed the benefit and risk of adding rivaroxaban for secondary prevention in patients with CAD, investigating the differences in treatment effects according to different baseline clinical presentations. The main findings of this meta‐analysis were as follows: (1) As published meta‐analyses, adding rivaroxaban to standard APT after CAD is associated with a reduction in the risk of ischemic events at the cost of a higher risk of bleeding; (2) In males the risk–benefit profile of rivaroxaban in addition to standard APT appears to be more favorable. Males have a reduced bleeding risk than females might be that they are less challenged (no menstruation or childbirth); (3) In patients < 65 years, in females, in patients with diabetes, those with mild to moderate impaired renal function, and region of Asia/other are associated with increasing in the risk of bleeding and with a nonsignificant reduction in the risk of ischemic events. Earlier studies found a higher rate of cardiovascular adverse outcomes and lower quality of life in females compared with males,^20^, ^21^ which might be related to females more often present with atypical symptoms and signs.^22^ Recent study demonstrated that females had higher rates of cardiovascular mortality and all‐cause mortality than male 1 year after acute myocardial infarction, as well as significantly poorer health status, even after adjustment for potential confounders, including baseline health status.^23^ Diabetes is a prothrombotic condition, exposing patients to a higher risk of cardiovascular events. This may explain why the risk of ischemic events in diabetic patients is non‐significantly decreased under rivaroxaban whereas it is significantly decreased in non‐diabetic patients. As well, the fact that rivaroxaban has partial renal elimination may explain why the risk of bleeding is particularly high in patients with moderate to severe renal impairment; (4) A comprehensive assessment of the risk of ischemic events and bleeding events is needed in patients ≥65 years, in males, in patients without diabetes, those with mild to moderate impaired renal function, America patients and Europe, because the use of rivaroxaban is associated with a significant decrease of the risk of ischemic events but at the cost of an increased risk of bleeding. (5) A similar risk of the primary efficacy endpoint was observed in patients with and without history of MI. In theory, patients with prior MI have a higher risk of ischemic events compared with those without a history of MI. In a pre‐specified subgroup analysis from the DAPT trial,^24^ the impact of prolonged therapy on major adverse cardiovascular events was more pronounced in participants with prior MI compared with those without an MI. However, our results were consistent with the subgroup analysis of the original studies. Such as the COMPASS trial, its subgroup analysis showed that the benefits of addition of low‐dose rivaroxaban to aspirin were consistent whether patients were within 2 years of myocardial infarction, 2–5 years after myocardial infarction, beyond 5 years, or never had an infarction. It may be related to the use of antiplatelet and anticoagulant dual channel antithrombotic therapy.

The optimal antithrombotic therapy aims to prevent thrombosis while avoiding hemorrhage. The ATLAS ACS 2‐TIMI 51 trial^7^ and COMPASS trial^9^ both indicated adding rivaroxaban to standard APT reduced the risk of MACE with higher risk of major bleeding, but without increasing the risk of fatal bleeding. In order to assess the net clinical benefit, the COMPASS trial^9^ made a net benefit analysis incorporating both ischaemic and bleeding events and indicated a significant net benefit in favor of rivaroxaban plus APT and deaths were reduced by 23%. Similarly, the ATLAS ACS‐TIMI 46 trial^14^ showed the rates of the net clinical outcome associated with rivaroxaban both 2.5 and 5 mg twice daily were directionally favorable.

Based on the results of published trials, the risk of bleeding with adding Xa factor inhibitors to APT was related to the dosage. The study ATLAS ACS‐TIMI 46^17^ is a dose‐escalation trial and 86.8% patients received rivaroxaban higher or equal to 10 mg per day (45.3% 10 mg daily, 22.1% 15 mg daily and 19.4% 20 mg daily). Results show the risk of the primary safety endpoint with rivaroxaban increased in a dose‐dependent manner. Similar results were confirmed in trials focused on Apixaban.^25^, ^26^ The study APPRAISE^25^ is a dose‐ranging trial of apixaban (in doses of 2.5 mg twice daily, 10 mg once daily, 10 mg twice daily, or 20 mg once daily) and the two higher‐dose arms (10 mg twice daily and 20 mg once daily) were discontinued because of excess total bleeding.

Antithrombotic therapy for patients with CAD is a long‐term management. The subgroup results are important to offer clinicians a more comprehensive picture of rivaroxaban as a therapeutic option in CAD. Clinicians should choose an optimal individualized regimen based on patients' bleeding risk and ischemic status, including age, sex, region, CAD presentations, APT regimens, as well as comorbid conditions such as heart failure, diabetes and renal insufficiency. Balancing the benefit and risk of adding rivaroxaban to APT for an individual patient is a clinical skill which can be supported with knowledge of these clinical characteristics and the available risk scores.

We acknowledge our meta‐analysis had several limitations. First, different studies had different primary efficacy endpoint and safety endpoint which might have influenced the results. Second, because of limited data, subgroup analysis of CAD presentations, APT regimens, dosage of rivaroxaban, and so forth were not performed. Third, given the limited number of studies included in the analysis, our findings should be confirmed with future research.

## 5 CONCLUSIONS

Rivaroxaban may provide an additional therapeutic choice for secondary prevention in patients with CAD. However, careful estimation the risk of cardiovascular ischemic and bleeding events using patient characteristics are critical to achieving net benefit.

## CONFLICT OF INTEREST

The authors declare no potential conflict of interest.

## Data Availability

Data sharing is not applicable to this article as no new data were created or analyzed in this study.
